# Ewing’s Sarcoma of the Head and Neck: Margins are not just for surgeons

**DOI:** 10.1002/cam4.1801

**Published:** 2018-11-17

**Authors:** Jebrane Bouaoud, Stephane Temam, Nathalie Cozic, Louise Galmiche‐Rolland, Kahina Belhous, Frederic Kolb, Francois Bidault, Stephanie Bolle, Sarah Dumont, Valerie Laurence, Dominique Plantaz, Marie‐Dominique Tabone, Perrine Marec‐Berard, Quentin Quassemyar, Vincent Couloigner, Arnaud Picard, Anne Gomez‐Brouchet, Marie‐Cécile Le Deley, Céline Mahier‐Ait Oukhatar, Natacha Kadlub, Nathalie Gaspar

**Affiliations:** ^1^ Unit of maxillofacial and Plastic Surgery Necker‐Enfants Malades Paris France; ^2^ Unit of Head and Neck Surgery Gustave Roussy Villejuif France; ^3^ Department of Biostatistics Gustave Roussy Villejuif France; ^4^ Department of Pathology Necker‐Enfants Malades Paris France; ^5^ University Paris Descartes Paris France; ^6^ Department of Pediatric Radiology Necker‐Enfants Malades Paris France; ^7^ Plastic and Reconstructive Surgery Department Gustave Roussy Villejuif France; ^8^ Department of Radiology Gustave Roussy Villejuif France; ^9^ Radiation Oncology Department Gustave Roussy Villejuif France; ^10^ Department of medical oncology Gustave Roussy Villejuif France; ^11^ Department of Medical Oncology Curie Institute Paris France; ^12^ Department of Pediatric Hematology‐Oncology University Hospital Centre of Grenoble Grenoble France; ^13^ Department of Paediatric Onco‐Haematology Armand Trousseau Hospital Paris France; ^14^ Department of Pediatric Oncology, Léon Bérard Cancer Center Institute for Paediatric Haematology and Oncology Lyon France; ^15^ Unit of Otolaryngology and Head and Neck Surgery Necker‐Enfants Malades Paris France; ^16^ Department of Pathology University Hospital of Toulouse Toulouse France; ^17^ Centre Oscar Lambret Lille France; ^18^ CESP, INSERM, Fac. de médecine ‐ Univ. Paris‐Sud Université Paris‐Saclay Villejuif France; ^19^ Department of Clinical Studies Unicancer Paris France; ^20^ Department of Oncology for Child and Adolescents Gustave Roussy Villejuif France

**Keywords:** Ewing’s sarcoma, head and neck, local treatment, long‐term sequeals, surgical margins

## Abstract

**Background, Methods:**

To describe the characteristics, treatments (systemic/local), and outcome (oncological/functional) of French patients with head and neck Ewing's sarcomas (HNES) registered in the Euro‐Ewing 99 (EE99) database. Specific patient‐level data were reviewed retrospective.

**Results:**

Forty‐seven HNES patients in the EE99 database had a median age of 11 years, 89% had bone tumors (skull 55%, mandible 21%, maxilla 11%), 89% had small tumors (<200 mL), and they were rarely metastatic (9%). Local treatment was surgery radiotherapy (55%), exclusively surgery (28%), or radiotherapy (17%). Metastatic relapses occurred in five patients with high relapse risk factors (metastasis at diagnosis, poor histological response, large tumors). Local progression/relapses (LR) after exclusive radiotherapy occurred in three patients with persistent extra‐osseous residue and in four patients considered R0 margins (postchemotherapy surgery, without postoperative radiotherapy [PORT]), reclassified by pathological review as R1a. Pathological review reclassified 72% of R0 margins: 11/18 to R1a and 2/18 to R2. Five patients had confirmed R0 margins after postchemotherapy surgery without PORT and had no LR Eight patients had R2 margins (initial surgery without previous chemotherapy, with PORT) and had no LR With a median follow‐up of 9.3 years, the 3‐year LR rate, EFS, and OS were 84.8%, 78.6%, and 89.3%, respectively. Among the 5‐year survivors, 88% had long‐term sequelae.

**Conclusion:**

To optimize HNES management, patients should be treated from diagnosis in expert centers with multidisciplinary committees to discuss treatment strategy (type of surgery, need for PORT) and validate surgical margins.

## 1 INTRODUCTION

Ewing's sarcoma (ES), although rare, is the second most common primary bone malignancy in children and adolescences^1^, ^2^ and is characterized by a specific transcript EWS/FLI‐1.^3^ Standard ES treatment consists of neoadjuvant/induction chemotherapy (neoCT), followed by local treatment (surgery and/or radiotherapy) combined with risk‐adapted consolidation/maintenance chemotherapy. This multidisciplinary approach is required as both systemic and local therapies are crucial.^4^

Head and neck ES (HNES) represent 1%‐15% of ES.^5^ The reported local control rates are low, between 71% and 81%.^6^, ^7^ Only a few studies have assessed the role of local treatment, which is particularly challenging and remains controversial.^8^ Surgery and radiotherapy have several objectives, including oncological (to allow satisfactory local disease control), functional (to preserve noble organs when possible), and esthetics (to maintain normal growth and adequate quality of life). Discussion of the role of each local therapy is essential, including the scheduling of local therapies with systemic treatment to maximize efficacy and minimize long‐term sequelae, especially in these young growing patients.

Our main objective was to describe patient and disease characteristics, the systemic and local treatments used, and the oncologic (relapses, survival), functional, and esthetic outcome, relative to the local treatment(s) administered in HNES patients.

## 2 PATIENTS AND METHODS

The study was supported by the French bone sarcoma group, GROUPOS. The EE99 trial (NCT00020566) was performed according to the ethical principles of the Declaration of Helsinki and good clinical practice guidelines. Written informed consents were obtained at enrollment from all patients or from their parents/guardians for those younger than 18 years of age.

### 2.1 Eligibility criteria

All French HNES patients aged <50 years, registered in the Euro‐Ewing 99 (EE99) trial, with a molecular diagnosis of ES were included in the study. Patients with intra‐orbital, cervical spine or intracranial origins of HNES were excluded.

### 2.2 Treatment according to the Euro‐Ewing 99 protocol

Initial tumor biopsy and extensive staging (local regional magnetic resonance imaging (MRI)/computed tomography (CT) scan, chest CT scan, bone scintigraphy, bone marrow biopsy/aspirates) were recommended before treatment. NeoCT, consisting of six courses of vincristine, ifosfamide, doxorubicin, and etoposide (VIDE),^9^ aimed to reduce the primary tumor volume and avoid/control metastases. Local treatment with surgery was recommended after neoCT, when feasible. Surgery was associated with postoperative radiotherapy (PORT) when resection margin was incomplete and/or when the histological response to neoCT was poor (≥10% viable residual cells).^10^ Exclusive radiotherapy was recommended for nonoperable primary tumors. Maintenance chemotherapy was allocated according to the ES risk of relapse. Patients with low‐risk localized disease, good histological response <10% viable cells (for operated primary tumors), or small initial tumor volumes <200 mL (in nonoperated primary tumors) were randomly assigned eight courses of vincristine and actinomycin D, combined with either ifosfamide (VAI) or cyclophosphamide (VAC).^11^ Patients with high‐risk localized ES, with or without isolated lung metastases, were randomly assigned high‐dose chemotherapy either busulfan/melphalan (Bu/Mel) or VAI plus bipulmonary radiotherapy for those with lung metastases.^12^, ^13^ Patients with multiple metastatic ES at diagnosis, other than isolated lung metastases, received Bu/Mel.^14^

### 2.3 The data extracted from the EE99 database

We analyzed the prospectively collected data extracted from the EE99 database including patient characteristics (age, gender), initial tumor presentations (histology, primary site, tumor volume, locoregional extension, metastatic status), systemic and local treatment modalities and scheduling, radiological and histological responses to treatments, and outcomes (relapse, death, late effects).

### 2.4 Multidisciplinary review of the patients’ data retrospectively collected

We then retrospectively collected and reviewed the charts, imaging, and surgical, pathological, and radiotherapy reports to refine data concerning local treatments and sequelae. The radiology (MRIs at diagnosis, before local treatment, after treatment, and at disease relapse); surgical and pathological reports (procedure and quality of the resection; Table 1)^15^; and the radiotherapy protocol (radiation field, dose delivered to tumor and surrounding organs) were each reviewed by two experts. These experts were selected from a multidisciplinary panel including surgeons (maxillofacial, otolaryngologist, plastic, neurosurgeons), radiologists, pathologists, and pediatric and radiation oncologists.

**Table 1:** Histological review of the 47 French HNES. Definition of the surgical margin classification according to Euro‐Ewing 99 and Euro‐Ewing 2012

	EE99	EE12
RO	Radical: clear margin	Clear margins 2 mm or more of normal tissue
R1	Marginal: macroscopically clear resection but microscopically margins are near the tumors	R1a: Resection in scar tissue, even clear of active tumor cells, within postchemotherapy fibrous reactive tissue (reactive fibrosis, edema, foamy macrophages, inflammatory cells)
		R1b: Resection in close contact with tumor (less than 2 mm, without any normal anatomical structure)
		R1c: Microscopical intralesional resection (viable tumor areas, in coagulative necrosis)
R2	Intralesional	On the base of surgeon report confirm by pathologist

In cases of margin R0, but fragmented resection or tumoral spreading the resection should be considered as R2 resection.

### 2.5 Acute complications and long‐term sequelae

Any event grade≥2, by common terminology criteria for adverse events (CTCAEv5.0), was collected.^16^ Acute complications were defined as any event occurring during or within three months after completing treatment; long‐term sequelae were defined as events that persisted for at least 5 years after treatment.^17^, ^18^ We classified these events by type (functional, aesthetic, psychological, social) and potential cause (surgery, radiotherapy, chemotherapy).

### 2.6 Statistical analysis

Overall survival (OS) and event‐free survival (EFS) were calculated from initiation of chemotherapy and estimated by Kaplan‐Meier method. EFS was defined as the delay from initiation of treatment until first failure (local progression/relapse, second malignancy, or death, whichever occurred first). Three‐year survival rates were estimated by Kaplan‐Meier method and presented with Rothman's 95% confidence intervals (CI). Median follow‐up was estimated by reverse Kaplan‐Meier method. The distributions of variables were compared between patient groups using Fisher's exact tests. A 5% significance level was used for all testing. All statistical analyses were performed with SAS® software version 9.4 (SAS Institute, Cary, NC).

## 3 RESULTS

**Figure 1:**
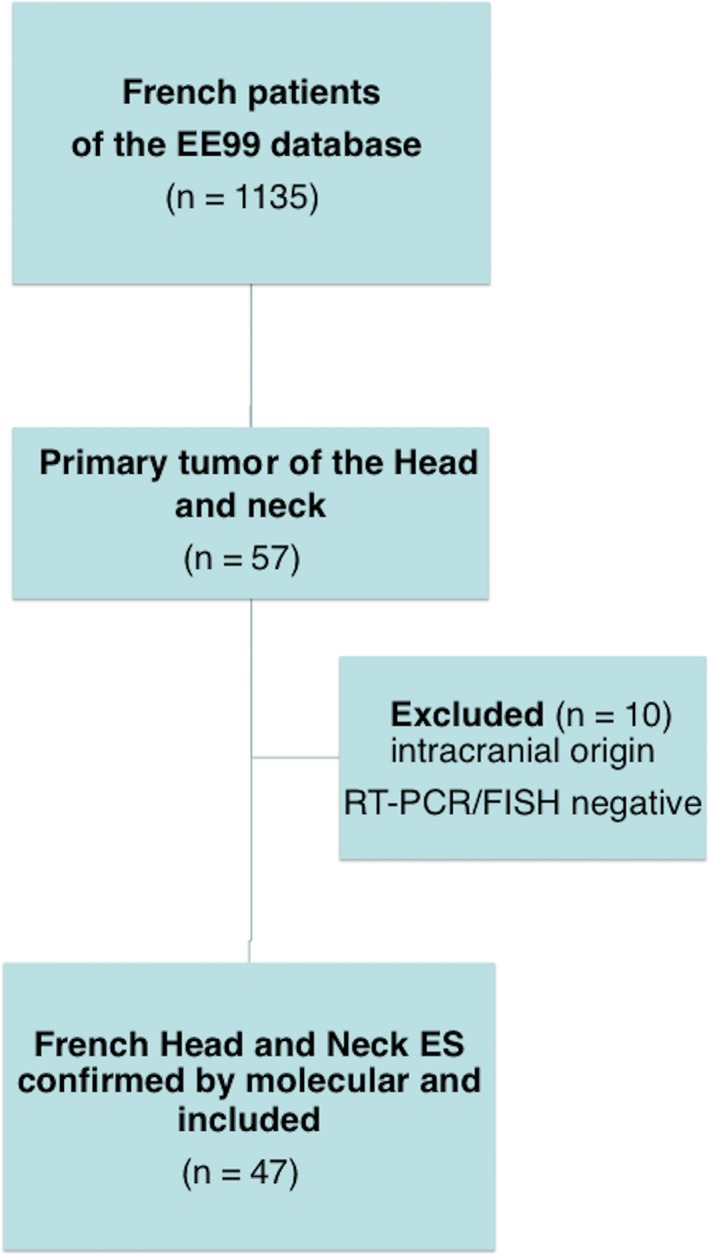
Flowchart. EE99, Euro‐Ewing 99 trial; ES, Ewing's sarcoma; FISH, fluorescence in situ hybridization; RT‐PCR, reverse transcription polymerase chain reaction

From September 1999 to December 2014, 1135 French patients were registered in the EE99 database, of which 57 patients had HNES primary tumors (Figure 1). Ten of these patients were excluded for inadequate primary tumor site (intra‐orbital, cervical spine, intracranial origin) or nonconfirmed ES molecular diagnoses. Finally, 47 patients were eligible: 4.1% of French ES patients in the EE99 database.

### 3.1 Patients and tumor characteristics

Twenty‐seven males and 20 females (sex ratio M/F = 1.4; Table 2) had a median age of 11 years (range, 1.2‐32) and either a prepuberty (n = 27; 57%) or intrapubertal status (n = 8; 17%). The median delay from initial symptom (mainly painful swelling) to diagnosis was 2 months (range, 7 days to 4 months).

**Table 2:** Patient/tumor characteristics of the 47 French HNES

Total (n = 47)	Patient characteristics: n (%)
Sex ratio M/F	27/20
Median age [range]	11 y [1.2‐32]
Pubertal status	Postpuberty 12 (26%)
Median delay symptom to diagnosis [range]	2 mo [7 d‐4 mo]
Primary origins	Osseous: 42 (89%) Extra osseous: 5 (11%)
Volume Size	Small: <200 mL: 42 (89%); median 70 mL [14‐900] <8 cm: 43 (91%); median 5 cm 8, 9
Locoregional lymph node N+	>10 mm: 12 (26%)
Metastasis at diagnosis	4 (9%)

**Figure 2:**
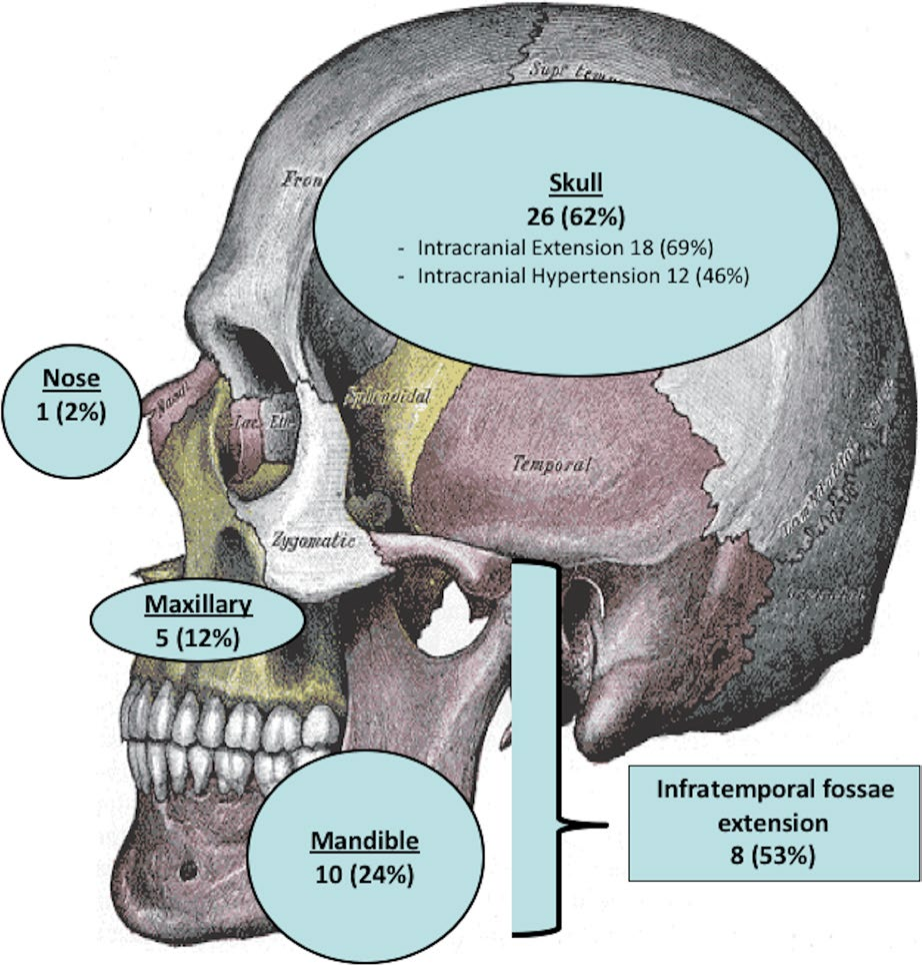
Characteristics of the 42 Osseous French HNES (n = 42/47)

Most primary tumors, 42/47 (89%), were osseous: located in the skull (n = 26), mandible (n = 10), maxillary (n = 5), and nose (n = 1) (Figure 2). The remaining five primary tumors were extra‐osseous: four subcutaneous and one intramuscular. Metastatic disease at diagnosis was rare in 4/47 (9%) (three lungs, one ribs), and most primary tumors 42/47 (89%) were small (initial tumor volume <200 mL).

Presentation and locoregional extension depended on the primary tumor location. Skull primary tumors originated from the vault (n = 13) and the base (n = 13) were complicated by an intracranial locoregional extension in 18 patients (69%) responsible for 12/18 cases of intracranial hypertension. Meningeal locoregional involvement was observed on imaging for nine patients (lumbar puncture results were not available). Intracranial venous sinus involvement was found in three cases. All these problematic extensions concerned mainly vault tumor. Mandible (n = 5/10) and maxillary (n = 3/5) primary tumors presented extension in the infratemporal fossa. Involvements of the orbit (n = 4) and skull base (n = 1) were seen with maxillary tumors.

Locoregional lymph nodes were considered pathological on imaging (smallest diameter >10 mm) without histological confirmation, for 12/47 patients (26%), with a higher relative frequency in mandible ES (n = 7/12). No histological vascular emboli or perinervous involvement was described in the pathological reports.

### 3.2 Diagnosis

All 47 patients had histological diagnoses confirmed either by fluorescence in situ hybridization (FISH) (EWSR1 gene rearrangement, n = 11) or by reverse transcription polymerase chain reaction (RT‐PCR) (EWS‐ETS fusion transcript, n = 36).

**Figure 3:**
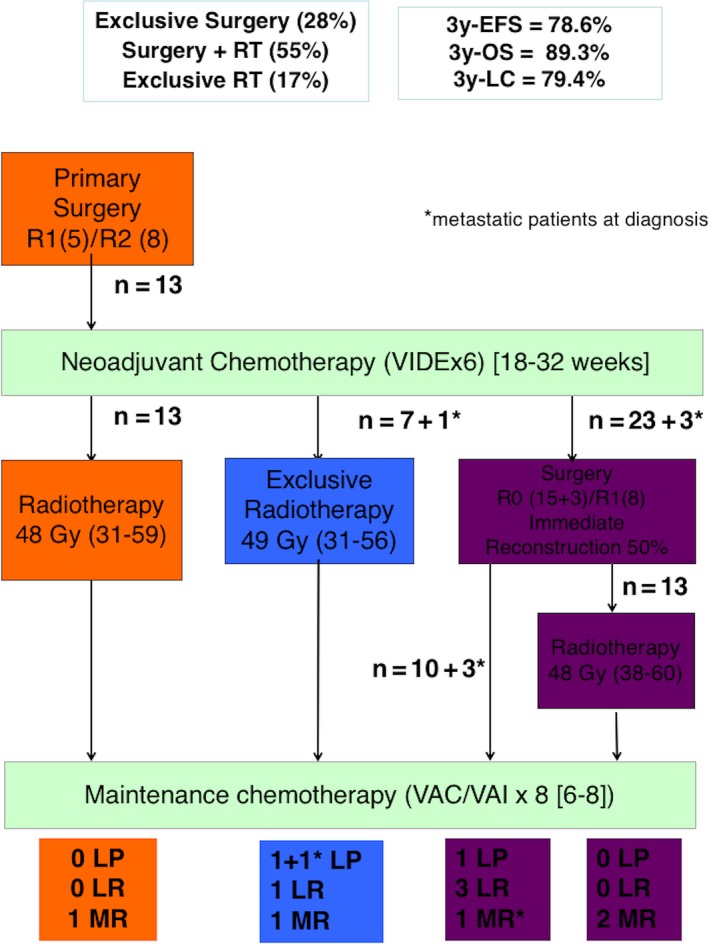
Treatment of the 47 French HNES. EFS, event‐free survival; LC, local control; LP, local progression; LR, local relapse; MR, metastatic relapse; OS, overall survival; R0, radical/clear margin; R1, marginal/macroscopically clear resection but microscopically margins are near the tumors; R2, intralesional; RT, radiotherapy; VAI/VAC, vincristine/actinomycin, and ifosfamide or cyclophosphamide; VIDE, vincristine/ifosfamide/doxorubicin/etoposide

The diagnoses were performed on biopsies before treatment in 34 patients (72%) (Figure 3). In addition, 13 patients had initial surgery without biopsy before any systemic treatment in an emergency context for six patients with intracranial hypertension or performed without suspicion of malignant tumor for seven patients (benign subcutaneous tumor suspected [n = 4], temporal bone infection, from chronic otitis, refractory to treatment [n = 2], and orbital cellulitis [n = 1]).

### 3.3 Systemic treatment according to EE99 trial

All patients received six courses of VIDE neoCT, 13 patients after initial surgery with a median delay of 24 days (range, 8‐55), and 34 patients after biopsy with a median delay of 13 days (range, 3‐37) (Figure 3). Radiological response after neoCT (using RECIST) was complete in six patients, partial (decrease ≥50% compared to baseline) in 19, and stable (decrease <50% compared to baseline without progressive disease) in 15. Histological responses were good (<10% of residual tumor cells) in 21/26 patients (81%) operated after neoCT. In the 26 patients operated after neoCT, maintenance chemotherapy started with a median delay of 18 days (range, 8‐64) from surgery (17/26 patients within three weeks after surgery). Forty‐seven patients received maintenance chemotherapy. Low‐risk localized ES (<10% residual viable cells [n = 19], initial tumor volume <200 mL treated by exclusive radiotherapy [n = 7], and initial primary surgery [n = 11]) received either VAI (n = 20) or VAC (n = 17). High‐risk localized ES (≥10% residual viable cells [n = 4] or initial tumor volume ≥200 mL with primary surgery [n = 2]) received VAI (n = 5) or Bu/Mel (n = 1). All of these patients received maintenance chemotherapy in accordance with their randomization (no modification of the protocol due to anticipated toxicity to busulfan). Patients with pulmonary metastases received VAC with lung radiotherapy (n = 1) or Bu/Mel (n = 2). The patient with distant bone metastasis received Bu/Mel.

### 3.4 Local treatment of the primary tumor

Thirteen patients had initial surgery without reconstruction (six intralesional resections and seven bone‐conserving surgeries or possible direct suture). All 13 received local PORT (mean dose 48 Gy, range 31‐59 Gy; Data S1).

Twenty‐six patients had surgery after neoCT, without immediate reconstruction in 13 (three intralesional resections, five surgeries of a small residual tumor volume not requiring reconstruction, two extra‐osseous lesions with direct suture of the operative site, three unspecified). PORT completed local treatment in 13/26 patients (median dose 48 Gy, range 38‐60 Gy; Data S1) for marginal resection (n = 8) or poor histological response to chemotherapy (40% of residual tumor cells) for one patient with complete resection (R0 margin). The remaining four patients had complete R0 resection and good histological response in the EE99 database, despite the absence of PORT indication in this situation in the EE99 trial. The reasons for PORT administration were explained neither in the database nor in the patient's chart.

Seven patients with inoperable skull base tumors and one patient/parent surgery refusal of a small maxillary tumor had exclusive radiotherapy (n = 8).

Overall, 39 patients (83%) were operated and 34 (72%) received radiotherapy.

### 3.5 Treatment of the locoregional extension

Initial radiological lymph node involvement disappeared after neoCT in six patients who did not receive specific lymph node treatment, and six patients had a lymphadenectomy, with no histological tumor involvement found and no additional radiotherapy.

Among the nine patients with meningeal involvement, one received exclusive whole‐brain irradiation and eight had surgery, including six with additional focal radiotherapy and two without radiotherapy. None had craniospinal irradiation.

### 3.6 Surgical margin

Among the 39 patients operated, the surgical margins in the EE99 database were wide/radical (R0) in 18 patients, marginal (R1) in 13, and intralesional (R2) in 8 (Table 3). All R2 resections occurred after initial surgery at diagnosis before chemotherapy (Data S2). R1 margins were more frequent after initial surgery (5/13) than after neoCT (8/26). R0 margin was observed in 18 patients only when operated after neoCT.

**Table 3:** Histological review of the 47 French HNES. Comparison of surgical resection margins for the 39/47 operated head and neck Ewing's sarcoma

euro‐EWING 2012 margin definition	euro‐EWING 99 margin definition		
	R0 (n = 18)	R1 (n = 13)	R2 (n = 8)
R0 (n = 5)	5		
R1 (n = 24)			
R1a	11	4	
R1b		1	
R1c		8	
R2 (n = 10)	2		8, all with initial surgery

Two patients were reclassified R2 despite R0 margins as the resection was fragmented (n = 1) or tumor break‐in (n = 1).

The pathological review according to EE2012 histological standardized report found a histological discordance rate of 72%: 13/18 R0 margins in the EE99 database were reclassified as R1a (n = 11; resection within postchemotherapy fibrous reactive tissue without viable tumor cells) or R2 margins (one fragmented resection, one tumor spreading during surgery) (Table 3).

### 3.7 Outcome

One patient who decided to stop treatment during maintenance chemotherapy developed a local relapse (LR) at 34 months after starting neoCT. This patient had exclusive surgery, considered as R0 resection with a good histological response to neoCT, and reclassified as R1a margin after the pathological review (Table 3). The 46 remaining patients completed treatment.

After treatment, 36/47 patients had complete remission (no radiological tumor residue) and 10/47 had a persistent residue. Among these ten patients, three had local disease progressions within 13‐16 months after starting neoCT, two in the radiation fields after local exclusive radiotherapy (one with initial meningeal involvement and one with rib metastasis at diagnosis), and one patient with localized disease reclassified from R0 to R1a margin (Table 3 and Figure 3). Of the 36 patients with complete remission at the end of treatment, three had a LR (median delay 24 months, range 18‐24) and five a metastatic relapse (median delay 33 months, range 17‐83). No regional lymph node or meningeal relapse was observed. One LR occurred in the radiation field of exclusive radiotherapy. Two LR occurred after exclusive surgery considered, in the EE99 database, as localized disease with wide margins and good histological response but reclassified as R1a after pathological review (Table 3). No LR was observed, with a median follow‐up of 11.8 years (range 3.3‐16.2), even in the absence of additional PORT, in the five patients who had exclusive surgery with R0 margins and good histological response (<10% viable residual cells) in both the EE99 database and pathological review (Table 3). Metastatic relapses occurred in one patient with initial bone metastasis, two with localized ES and very poor histological response (>70% viable tumor cells), and one with a large initial tumor volume operated before any chemotherapy with R1c margin and PORT. Finally, a patient had a small nonoperable tumor and received exclusive radiotherapy as local treatment.

No second malignancy was declared in the EE99 database. However, one patient with a histologically proven relapse in the lung 6 years after HNES diagnosis developed a local tumor in the radiation field 8 years after diagnosis and following an R1c resection. No histology was performed, but this evolution could possibly be a radiotherapy‐induced second cancer.

Eight patients died, all of HNES progression between 1.5 and 6.5 years after starting treatment.

The median survival for the 12 patients with events was 3.2 years (95%CI: 1.9y‐NA).

**Figure 4:**
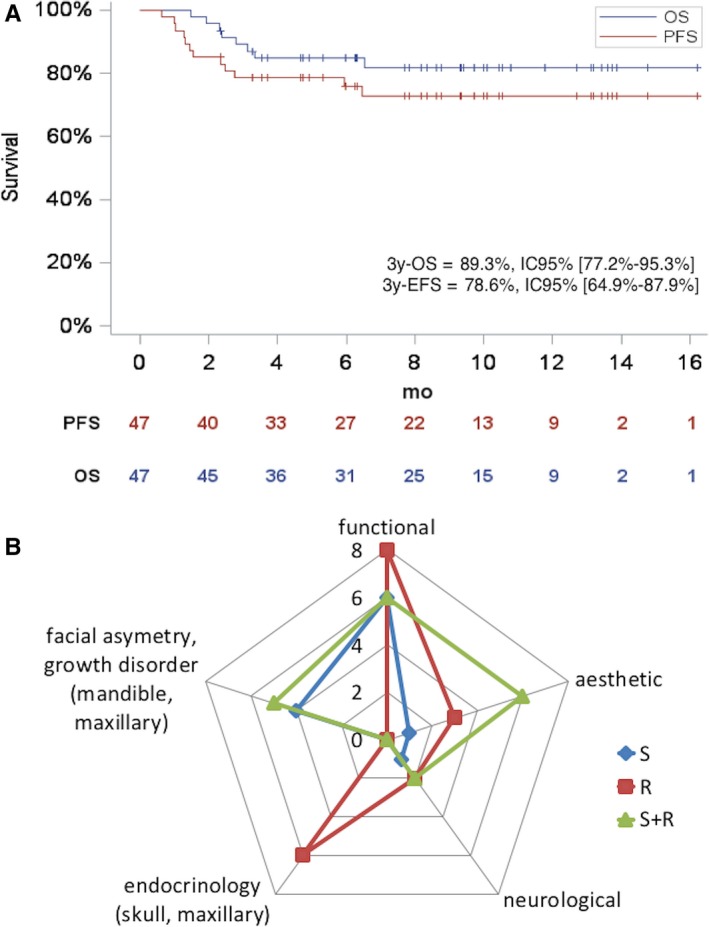
Outcome of the 47 French HNES. A, 3‐years event‐free survival and overall survival for the 47 head and neck Ewing's sarcoma. B, Long‐term sequelae according to local treatment in head and neck Ewing's sarcoma for the n = 40 patients alive after at least 5 years from initial treatment. CI, confidence interval; EFS, event‐free survival; OS, overall survival; R, radiotherapy; S, surgery

Overall, with a median follow‐up of 9.3 years (range 1.5‐16.2), for the all HNES population, the 3‐year local control, EFS, and OS rate were 79.4%, (95%CI: 65.2‐88.7), 78.6% (95%CI: 64.9‐87.9), and 89.3% (95%CI: 77.2‐95.3), respectively (Figure 4A). For the 43 localized HNES, the 3‐year local control, EFS, and OS rate were 88.8% (95%CI: 74.4‐95.6), 81.3% (95%CI: 67.2‐90.2), and 90.6% (95%CI: 78.2‐96.3), respectively.

### 3.8 Local long‐term sequelae

Among the 40 patients alive after at least 5 years from initial treatment, 88% developed long‐term sequelae (n = 35): functional (n = 20), growth abnormalities with face asymmetry (n = 9), aesthetic (n = 10), endocrine disorders (n = 6), and neurological (n = 4) or psychosocial impairments (n = 9) (Figure 4B). Their occurrence depended on the primary tumor location, patient's age, local treatment modalities (surgery and/or radiotherapy), and the possibility of immediate reconstruction. Almost all patients (n = 23/25, 92%) treated by surgery and radiotherapy developed sequelae.

## 4 DISCUSSION

We confirmed that HNES are rare (4.1% of all ES) (Data S3),^6^, ^7^, ^19^, ^20^, ^21^, ^22^ arising mainly from bones (skull, mandible, maxillary)^23^ in children/adolescents with persistent growing potential (median age 11 years; only 26% postpuberty). Most HNES patients have low ES relapse risk factors 24:91% with nonmetastatic tumors, 89% with small primary tumors at diagnosis, and 81% with good histological response to chemotherapy and a favorable outcome^25^ (3y‐EFS and OS of 78.6% and 89.3%, respectively). However, for the minority of HNES patients with high ES relapse risk factors (metastatic disease and/or poor histological response), the outcome is impaired by metastatic relapses, as in other ES localizations.^26^, ^27^ The main issues are the occurrence of LR (3y‐LR rate 84.8%) and long‐term sequelae (88%). Patients with LR usually died from disease progression within three years of LR Consequently, local HNES treatment is challenging in terms of local disease control and limiting long‐term sequelae in these children/adolescents still with growth potential.^28^, ^29^

Other than the German Society for Pediatric Hematology and Oncology (GPOH) series of 51 patients,^7^ our study is the largest HNES published series with a homogenous systemic treatment, general strategy, and local treatment indications (according to the EE99 trial) and with a long‐term follow‐up (9.3 years). Although previous series showed no significant differences in terms of outcome (EFS/OS) with the different local treatment modalities,^7^ our series assessed these different local treatment strategies, using prospective and retrospective data, in terms of local control, survival, and long‐term sequelae. A strength of our study is the retrospective review of key data from the patients’ medical files by experts in radiology, pathology, surgery, and radiotherapy/pediatric/medical oncology that refined the data extracted from the EE99 database. This review, since retrospective, may be considered as a weakness. However, the review identified major discrepancies that would have been ignored by the analysis of only the prospective database.

Radiological review revealed that 25% of patients had regional lymph node and 19% had meningeal extensions; thus, these were not so rare and possibly poorly estimated at diagnosis. None of the regional lymph node extensions, diagnosed using the lymph node size on MRI, was confirmed histologically or by positron‐emission tomography (PET) scan. Similarly, none of the meningeal extensions was confirmed by lumbar puncture. Nevertheless, no lymph node or meningeal relapses were observed suggesting that current management is adequate. Extensive lymph node surgery or radiotherapy, as well as craniospinal irradiation, appears not to be essential for regional extension control. Avoiding these procedures may reduce the occurrence of long‐term sequelae.

Radiological and radiotherapy review revealed that patients treated by exclusive radiotherapy had dismal prognoses (3/8 local events and death), as previously described,^7^ especially when an extra‐osseous residue persisted after treatment (all three patients relapsed). Thus, we recommend that all patients be operated, whenever possible, especially when an extra‐osseous tumor residue persists. In these cases, referral to expert centers to discuss optimal surgery is required.

The pathological review showed a 72% discordance concerning the quality of surgical margins when initially considered as R0, 11 being reclassified as R1a margin and two as R2 margins. These incorrect classifications may have severe consequences in terms of treatment options, LR, and the occurrence of long‐term sequelae. Indeed, all local events (4 LR/progression) after neoCT and surgery occurred in patients with R0 margins reclassified as R1a and who did not receive PORT. Thus, PORT should be administered in patients with R1a margins, even if residual viable tumor cells are not present. PORT may prove effective for patients with intralesional surgery (R2), as no patient with R2 margin resection who received PORT experienced a LR The surgery/PORT combination compared to surgery alone as local treatment showed no clear excess of long‐term sequelae, but the number of patients might not be sufficient to observe a difference. No second cancers were observed after a median follow‐up of 9.3 years. However, the theoretical increase in risk with radiotherapy of growth sequelae and second cancer in these growing children/adolescents may become evident with longer follow‐up.^16^, ^30^, ^31^ Half of the R2 procedures occurred during clinically urgent initial surgery. Attempts must be made to avoid these urgent unplanned surgeries, and biopsies should be considered. In contrast, all five patients with R0 margins by planned surgery after neoCT, confirmed by our pathological review, and with good response to chemotherapy did not experience LR, even without PORT. Thus, planned postchemotherapy HNES surgery with true R0 margins and good histological response to chemotherapy may not require PORT. In these growing patients, avoiding PORT by a R0 surgery might spare these patients the long‐term risk induced by radiotherapy.

Consequently, all actors involved in HNES management, including medical, pediatric, and radiation oncologists, and not only surgeons and pathologists, should question the surgical and pathological reports and properly define surgical margins that condition the use of PORT. To improve the quality of surgical margin reporting, standardized surgical and pathological reports^15^ have been implemented in the EE2012 trial (EudraCT‐2012‐002107‐17). However, multidisciplinary discussions are essential after surgery to evaluate the need of PORT and to balance the importance of local control with the risk of long‐term sequelae. More importantly, these discussions concerning the overall management of HNES should take place early, at diagnosis, to anticipate local treatments with two objectives: 1‐to avoid initial R2 surgery when feasible and 2‐to balance the benefit/risk of a R0 procedure after neoCT compared to a R1 surgery associated with PORT. To lower the current 55% of combined surgery‐radiotherapy used, we need to obtain more R0 margins accurately classified, to achieve better local control and avoid radiotherapy, thus minimizing long‐term sequelae without jeopardizing the use of systemic chemotherapy. This requires expert surgical teams^19^, ^32^, ^33^ to ensure quality resections and adequate reconstruction procedures and may require patient referral to competent surgeons in expert centers, with a wider expertise in HNES management. When R1 resections are unavoidable, PORT is required^34^, ^35^ and pediatric radiotherapy expertise is necessary to minimize the risks of sequelae in these children/adolescents whose growth potential might be altered and with the added risk of developing second cancers throughout their lives.^36^, ^37^

## CONFLICT OF INTEREST

Nothing to declare. This research did not receive any specific grant.
