# Supplementary material for: Ewing’s Sarcoma of the Head and Neck: Margins are not just for surgeons
Source: Cancer Med. 2018 Nov 17;7(12):5879–88. doi: 10.1002/cam4.1801 (PMC6308064; doi:10.1002/cam4.1801)
Supplement: Supplementary file 1 [file CAM4-7-5879-s001.docx]

**Supplementary Data HEAD AND NECK EWING SARCOMA (n=47)**

Supplementary data 1. Radiotherapy protocol for Head end Neck Ewing Sarcoma (n=47). (RT: radiotherapy)

| RT (n=34) | Median dose  Range (-) | Fractionation per dose | Type tumor  Indication RT |
| --- | --- | --- | --- |
| RT in complement of primary surgery (n=13) | 48 Gy  (31-59 Gy) | 1,8 Gy by session | Skull 10; sub-cutaneous n=2; infra temporal fossae n=1;  n=13 tumor operated before any diagnosis, all with positive surgical margins. |
| Post operative RT (n=13) | 48 Gy  (38-60Gy) | 1,8 Gy by session | Skull n=4; mandible n=4; maxillary n=4; sub-cutaneous n=1;  n=8 marginal resection margin (R1)  n=1 poor response to chemotherapy |
| Exclusive RT (n=8) | 47 Gy  (31-54Gy) | 1,8 Gy by session | Skull n=7 (base n=3; vault n=4)  Maxillary n=1  Infeasible surgery because: very extensive tumor  and / or  reaching skull base  and / or  risk of mutilating surgery |

Supplementary data 2. Surgical resection margin of Head and Neck Ewing Sarcoma according EE99 definition. (EE99: euro-EWING 1999)

| N= 39 Head and Neck ES operated | | Primary surgery (n=13) | Post chemotherapy surgery (n=26) |
| --- | --- | --- | --- |
| EE99 | R0 (radical) | 0 | 18 |
|  | R1 (marginal) | 5 | 8 |
|  | R2 (intralesional) | 8 | 0 |

Supplementary data 3. Literature review (MFU: median follow-up; OS: overall survival; EFS: event free survival; IESS: intergroup Ewing’s sarcoma study; CT: chemotherapy; S: surgery; RT: radiotherapy; NA: not available; m: months ; y: years; ES: Ewing sarcoma; PNET: primitive neuroectodermal tumor; RMS: Rhabdomyosarcoma; MMT: Malignant Mesenchymal Tumor)

| **Study author years of publication** | **Treatment protocol** | **Inclusion years** | **Number evaluated subject (% M+)** | **HN ES prevalence** | **Median age [range]**    **Sex ratio** | **Primary involved sites (n)** | **Local treatment n (%)** | **Histological Margin** | **Good histological response to CT** | **Local control rate** | **MFU** | **3-y OS; 3-y EFS** | **5-y OS; 5-y EFS** |
| --- | --- | --- | --- | --- | --- | --- | --- | --- | --- | --- | --- | --- | --- |
| Siegal  1987 | IESS therapy protocol: CT +/- R | 1972- 1987 | 29 (7%) | 4% | 11 y  [3-23]  M/F=1,2 | Skull (11)  Cervical vertebrae (7)  Mandible (6)  Maxillary (4) | R 15 (52%)  S+R 14(48%) | NA | NA | NA | 56m | 80%; NA | NA |
| Allam  1999 | CT+ R (58%)  S + post op CT (21%)  Other protocols (21%)  At all 92% CT (VAC 45%; VAC + IEP 32%; VAIA 23%) | 1975-1996 | 24  (13%) | 9% | 16,5 y  [2-33]  M/F=2,4 | Maxillary (9)  Mandible (6)  Orbit (4) | R 16 (67%)  S 4 (16,5%)  S+R 4 (16,5%) | R negative 2 (25%) | NA | 71% | 41m | NA | 53%; 30% |
| Abdel Rahman 2010 | At all 95% of uniform CT +/- S and/or R | 1997-2008 | 20 (30%) | 7% | 11.5 y [5m-22y]  M/F=1 | Mandible (9)  Neck mass (4) clavicle mass (3) | R 8 (40%)  S 2 (10%)  S+R 5(25%) | R negative 4 (57%) | NA | NA | 36m | 50%; 67% | NA |
| Berger  2013 | Induction CT +  S and/or R + consolidation CT (8 Bu/mel) | 1980-2009 | 26: 21 ES + 5 PNET (19%) |  | 10y  [1,6-18]  M/F=1,6 | NA | R 9(35%)  S 5 (19%)  S+R 8(31%)  Unknown 4 (15%) | Histological and/or radiological good response:  7 (27) | 41% | NA | 59 m | NA | NA  10y OS and ES: 63% and 52% |
| Biswas  2014 | Uniform Induction CT +  S and/or R + adjuvant CT | 2003-2011 | 35 (9%) | 9% | 12y  [1-43y]  M/F=1,1 | Maxillary (14)  Mandible (7)  Orbit (5) | S 1(3%)  R 23(66)  S+R 8 (23) | R negative (78%) | NA | 74 (at 5y) | 58m | NA | 68%; 55% |
| Grevener 2015 | EE99: induction CT (6VIDE)+  S and/or R + consolidation CT (7 VAI or 7 VAC or Bu/mel) | 1999-2009 | 51 (14%) | 3% | 11.6y  [3m-66y] M/F=1,1 | Skull (23)  Other (7)  Maxilla (7) Mandible (6) | S 17 (33%)  R 9 (18%)  S+R 23 (45%) | EE99:  R0 13 (36%)  R1 11 (31%)  R2 10 (28%) | 80% | NA | 40m | 77%; 67% | NA |
| Qureshi 2016 | Induction CT (2VIE+ 2VAC) +  S and/or R + maintenance CT (4 VAC, 2 VIE, 4 VCD) | 2005-2015 | 21 (0) | 4% | 11.6y  [5-17]  M/F=1,1 | Maxilla (13)  Mandible (8) | S 5(24%)  R 4(19%)  S+R 12(57%) | R negative 14 (82%) | 50% | 80% | 36m | 68%; 64% | NA |
| Bouaoud 2018 | EE99: induction CT (6VIDE)+  S and/or R + consolidation CT (7 VAI or 7 VAC or Bu/mel) | 1999- 2014 | 47 (9%) | 4.1% | 11 y  [1.2-32]  M/F=1,4 | Skull (26)  Mandible (10)  Maxillary (5) | S 13(28%)  R 8 (17%)  S+R 26 (55%) | EE99:  R0 19 (49%)  R1 12 (31%)  R2 8 (20%) | 85% | 79% | 112 m | 89%; 79% | NA |
| Others head and neck tumors | | | | | | | | | | | | | |
| Orbach 2016  (HN RMS) | MMT-84, MMT-89, and MMT-95 protocols  CT +/- S and/or R | 1984-2004 | 140 (NA) | 9,3% of all RMS | 5y  [0.2–17.9]  M/F=1,1 | Superficial face 46%  Oral cavity (21%)  Neck (19%)  Salivary glands (14%) | S 32 (23%)  R 27 (19%)  S+R 33 (24%) | R0  9 (14%)  R1 27(42%)  R2 29 (45%) | 51% | 96%, 49% relapsed (loco regionally 91%) | 120m | NA | 74.7%; 48.9% |
